# Bisphenol AF Is a Full Agonist for the Estrogen Receptor ERα but a Highly Specific Antagonist for ERβ

**DOI:** 10.1289/ehp.0901819

**Published:** 2010-04-28

**Authors:** Ayami Matsushima, Xiaohui Liu, Hiroyuki Okada, Miki Shimohigashi, Yasuyuki Shimohigashi

**Affiliations:** 1 Laboratory of Structure-Function Biochemistry, Department of Chemistry, Research-Education Centre of Risk Science, Faculty and Graduate School of Sciences, Kyushu University, Fukuoka, Japan; 2 Division of Biology, Department of Earth System of Science, Faculty of Science, Fukuoka University, Fukuoka, Japan

**Keywords:** bisphenol A, bisphenol AF, endocrine disruptor, estrogen receptors, receptor antagonist, receptor binding

## Abstract

**Background:**

Bisphenol AF has been acknowledged to be useful for the production of CF_3_-containing polymers with improved chemical, thermal, and mechanical properties. Because of the lack of adequate toxicity data, bisphenol AF has been nominated for comprehensive toxicological characterization.

**Objectives:**

We aimed to determine the relative preference of bisphenol AF for the human nuclear estrogenic receptors ERα and ERβ and the bisphenol A–specific estrogen-related receptor ERRγ, and to clarify structural characteristics of receptors that influence bisphenol AF binding.

**Methods:**

We examined receptor-binding activities of bisphenol AF relative to [^3^H]17β-estradiol (for ERα and ERβ) and [^3^H]bisphenol A (for ERRγ). Functional luciferase reporter gene assays were performed to assess receptor activation in HeLa cells.

**Results:**

We found that bisphenol AF strongly and selectively binds to ERs over ERRγ. Furthermore, bisphenol AF receptor-binding activity was three times stronger for ERβ [IC_50_ (median inhibitory concentration) = 18.9 nM] than for ERα. When examined using a reporter gene assay, bisphenol AF was a full agonist for ERα. In contrast, it was almost completely inactive in stimulating the basal constitutive activity of ERβ. Surprisingly, bisphenol AF acted as a distinct and strong antagonist against the activity of the endogenous ERβ agonist 17β-estradiol.

**Conclusion:**

Our results suggest that bisphenol AF could function as an endocrine-disrupting chemical by acting as an agonist or antagonist to perturb physiological processes mediated through ERα and/or ERβ.

Bisphenol AF (also referred to as hexafluoro- bisphenol A) is a homolog of bisphenol A (BPA) (Figure 1). Bisphenol AF has a symmetrical chemical structure of HO–C_6_H_4_–C(CF_3_)_2_–C_6_H_4_–OH and is designated as 1,1,1,3,3,3-hexafluoro-2,2-bis(4-hydroxyphenyl)propane by IUPAC (International Union of Pure and Applied Chemistry) nomenclature. Bisphenol AF–containing polymers such as polycarbonate copolymers, polyimides, polyamides, and polyesters are used in high-temperature composites, electronic materials, and gas-permeable membranes. Bisphenol AF is also used in many other specialty polymer applications, including plastic optical fibers and waveguides. Although industrial production of bisphenol AF seems to be increasing considerably, no data are available on annual production or concentrations of bisphenol AF in environmental substrates.

In 2008, the U.S. National Institute of Environmental Health Sciences nominated bisphenol AF for comprehensive toxicological characterization based on the lack of adequate toxicity data [National Toxicology Program (NTP) 2008a]. In this nomination report, the NTP noted concern regarding potential exposure of the general population to bisphenol AF. Structural dissimilarities between bisphenol AF and BPA are determined by the presence of a trifluoromethyl (CF_3_) or methyl (CH_3_) group, respectively. The potential toxicity of bisphenol AF is of concern in part because its CF_3_ group is much more electronegative (and potentially reactive) than is the CH_3_ group of BPA.

Various “low-dose effects” of BPA have recently been reported *in vivo* for reproductive organ tissues in mice and rats. For example, *in utero* exposures to very low levels of BPA have been shown to increase the size and weight of the fetal mouse prostate (Gupta 2000; Nagel et al. 1997), and low-dose exposures have also been reported to decrease daily sperm production and fertility in male mice (Gupta 2000; vom Saal et al. 1998). Many lines of evidence have recently indicated that low doses of BPA affect the central nervous system as well (vom Saal and Welshons 2005; Welshons et al. 2003, 2006). All of these low-dose effects of BPA have been attributed to effects on steroid hormone receptors such as estrogen receptor (ER) and androgen receptor (AR) (Welshons et al. 2003; Xu et al. 2005). In the report by the NTP (2008b) on the potential for BPA exposure to affect human reproduction or development, “some concern” was indicated as the level of concern for potential effects on the brain, behavior, and the prostate gland.

BPA exhibits extremely weak binding activity for ER and AR. Based on the idea that BPA may interact with nuclear receptors (NRs) other than ER and AR, we screened a series of NRs and eventually discovered estrogen-related receptor γ (ERRγ) as the BPA target receptor (Takayanagi et al. 2006). BPA binds to ERRγ very strongly [dissociation constant (*K*_d_) = 5.5 nM] with high constitutive basal activity (Liu et al. 2007; Okada et al. 2008; Takayanagi et al. 2006). Strong binding of BPA to ERRγ was further demonstrated by direct X-ray crystallographic analysis of this complex (Matsushima et al. 2007, 2008). Moreover, using real-time PCR (polymerase chain reaction), we recently demonstrated that human *ERR*γ mRNA is expressed abundantly in the placenta, prostate, and fetal brain (Takeda et al. 2009).

Our efforts to explore the target receptor of BPA suggested that it is essential to examine endocrine chemicals for interactions with all 48 human NRs. We previously reported that bisphenol AF binds to ERα more strongly than does BPA, and that the receptor selectivity of bisphenol AF is seven times higher for ERα than for ERRγ (Okada et al. 2008). There are two subtypes of estrogen receptors, ERα and ERβ, with distinctly different physiological distributions and functions. Because effects of a number of chemicals have been reported to differ between ERα and ERβ (Harris et al. 2003; Manas et al. 2004), it is important to examine the effects of bisphenol AF on both ERs. In the present study, we evaluated the binding activity and functional biological activity of bisphenol AF for ERβ and found that bisphenol AF is a potent ligand that functions as an antagonist on ERβ.

## Materials and Methods

### Test compounds

We obtained 17β-estradiol (CAS no. 50-28-2; 98.9%) from Research Biochemicals International (Natick, MA, USA), and BPA (CAS no. 80-05-7; purity 99%) and bisphenol AF (CAS no. 1478-61-1; purity 99%) from Tokyo Kasei Kogyo Co. Ltd. (Tokyo, Japan). 4-Hydroxytamoxifen (4-OHT; CAS no. 68047-06-3; purity 98%) and 2,2-bis(*p*-hydroxyphenyl)-1,1,1-trichloroethane (HPTE) were obtained from Sigma-Aldrich Inc. (St. Louis, MO, USA).

### Preparation of glutathione S-transferase–(GST)-fused NR ligand-binding domain (LBD) protein

cDNA clones of ERα and ERβ were purchased from OriGene Technologies, Inc. (Rockville, MD, USA). GST-fused receptor LBDs expressed in *Escherichia coli* BL21α (GST-ERα-LBD, GST-ERβ-LBD, and GST-ERRγ-LBD) were purified on an affinity column of glutathione-Sepharose 4B (GE Healthcare BioSciences Co., Piscataway, NJ, USA) followed by gel filtration on a Sephadex G-10 column (15 × 10 mm; GE Healthcare BioSciences).

### Radioligand binding assays for saturation binding

We conducted the saturation binding assays for ERα and ERβ essentially as reported by Nakai et al. (1999) using tritium-labeled ligand [^3^H]17β-estradiol (5.96 TBq/mmol; GE Healthcare UK Ltd., Buckinghamshire, UK). Receptor protein GST-ERα-LBD or GST-ERβ-LBD (0.3 nM) was incubated with increasing concentrations of [^3^H]17β-estradiol (0.1–30 nM) in a final volume of 100 μL binding buffer (10 mM Tris, 1 mM EDTA, 1 mM EGTA, 1 mM sodium vanadate(V), 0.5 mM phenylmethylsulfonyl fluoride, 0.2 mM leupeptin, 10% glycerol; pH 7.4). Nonspecific binding was determined in a parallel set of incubations that included 10 μM nonradiolabeled 17β-estradiol. After incubation for 2 hr at 20°C, free radioligand was removed by incubation with 0.4% dextran-coated charcoal (Sigma-Aldrich Inc.) in phosphate-buffered saline (PBS; pH 7.4) for 10 min on ice and then centrifuged for 10 min at 15,000 rpm.

We performed the saturation binding assay for ERRγ as reported previously (Okada et al. (2008) using [^3^H]BPA (5.05 TBq/mmol; Moravek Biochemicals, Brea, CA, USA). Specific binding of tritium-labeled ligand was calculated by subtracting the nonspecific binding from the total binding. Receptor proteins that were expressed and purified were evaluated in a saturation binding assay to estimate *K*_d_ and receptor density (*B*_max_), and only good-quality preparations with appropriate *K*_d_ and *B*_max_ were used for competitive receptor-binding assays.

### Radioligand binding assays for competitive binding

Bisphenol AF, BPA, 17β-estradiol, and 4-OHT were dissolved in 0.3% DMSO in 1% bovine serum albumin (BSA; a blocker of nonspecific adsorption to the reaction vessels). HPTE was tested as a reference compound that acted as an ERα agonist and an ERβ antagonist. These chemicals were examined for their ability to inhibit the binding of [^3^H]17β-estradiol (5 nM in final) to GST-ERα-LBD (26 ng) and GST-ERβ-LBD (26 ng). The reaction mixtures were incubated overnight at 4°C, and free radioligand was removed with 1% dextran-coated charcoal by filtration. Radioactivity was determined on a liquid scintillation counter (TopCount NXT; PerkinElmer Life Sciences Japan, Tokyo, Japan). We calculated the half-maximal inhibitory concentrations (IC_50_) for 17β-estradiol from dose–response curves obtained using the nonlinear analysis program ALLFIT (DeLean et al. 1978). Each assay was performed in duplicate and repeated at least five times. For reconfirmation, we also performed the binding assay for ERRγ using [^3^H]BPA (5 nM final concentration) and GST-ERRγ-LBD (26 ng).

### Luciferase reporter gene assay

HeLa cells were maintained in Eagle’s minimum essential medium (MEM; Nissui, Tokyo, Japan) in the presence of 10% (vol/vol) fetal bovine serum at 37°C. For luciferase assays, HeLa cells were seeded at 5 × 10^5^ cells per 6-cm dish for 24 hr and then transfected with 4 μg reporter gene (pGL3/3xERE) and 3 μg of ERα or ERβ expression plasmid (pcDNA3/ERs) by Lipofectamine Plus reagent (Invitrogen Japan, Tokyo, Japan) according to the manufacturer’s protocol. Approximately 24 hr after transfection, cells were harvested and plated into 96-well plates at 5 × 10^4^ cells/well. The cells were then treated with varying doses of chemicals diluted with 1% BSA/PBS (vol/vol). After 24 hr, luciferase activity was measured with the appropriate reagent using a Luciferase Assay System (Promega, Madison, WI, USA) according to the manufacturer’s instructions. Light emissions were measured using a Wallace 1420 ARVOsx multilabel counter (PerkinElmer). Cells treated with 1% BSA/PBS were used as a vehicle control. Each assay was performed in triplicate and repeated at least three times. The assay for ERRγ was carried out as previously reported (Okada et al. 2008).

To measure the antagonistic activity of bisphenol AF for ERβ, we examined four concentrations (0.01, 0.1, 1.0, and 10 μM) of bisphenol AF for a serial concentration of 17β-estradiol (10^−12^ to 10^−5^ M in the final solution). Also, a serial concentration of bisphenol AF (10^−12^ to 10^−5^ M in the final solution) was assayed in the presence of 10 or 100 nM concentrations of 17β-estradiol, which normally elicit full activation of ERβ.

## Results

### Strong binding activity of bisphenol AF to ERβ receptor

We selected receptor protein preparations suitable for the competitive receptor-binding assay based on Scatchard plot analyses of saturation-binding assays. Receptor populations with the appropriate dissociation constant (*K*_d_) and receptor density (*B*_max_) were used for each radioligand receptor-binding assay. Because all of the NRs are secreted protein preparations, observed *B*_max_ values were comparable with those calculated from their molecular weight.

BPA was a very weak ligand for ERα (IC_50_ = 1,030 nM) based on its ability to inhibit [^3^H]17β-estradiol binding (Figure 2A, Table 1), as we previously reported (Okada et al. 2008). In the present study, we confirmed that BPA is also a very weak ligand for ERβ (IC_50_ = 900 nM; Figure 2B, Table 1), indicating comparable interactions of BPA with ERα and ERβ despite the subtle structural differences between these ERs. In contrast, bisphenol AF was 20 times more potent than BPA as a ligand for ERα (IC_50_ = 53.4 nM; Figure 2A, Table 1) and was approximately 48 times more potent for ERβ (IC_50_ = 18.9 nM; Figure 2B, Table 1). This high binding activity for ERβ suggests that the binding pocket of ERβ possesses specific structural elements that interact much more favorably with the CF_3_ groups of bisphenol AF than with the CH_3_ groups of BPA. We also assayed HPTE, an analog of BPA and bisphenol AF with the CCl_3_ group. HPTE was almost equipotent to bisphenol AF in the assays for both ERα and ERβ (Table 1), but approximately 10 times more potent than bisphenol AF for ERRγ.

### Receptor-binding selectivity of bisphenol AF and BPA

We used the IC_50_ values shown in Table 1 (from the competitive receptor-binding assay for nuclear ERα, ERβ, and ERRγ) to estimate receptor selectivity ratios for BPA and bisphenol AF (Table 2). The results indicate that BPA is exclusively selective for ERRγ, being 90–100 times more active for ERRγ than for ERα or ERβ. In contrast, bisphenol AF receptor binding is much more selective for ERα and ERβ than for ERRγ (6.70 times more selective for ERα than for ERRγ and 18.94 times more selective for ERβ than for ERRγ; Table 2). Bisphenol AF binding is also about three times more potent for ERβ than for ERα.

### Differential effects of bisphenol AF in the reporter gene assay

We next examined reporter gene activity after bisphenol AF exposure in HeLa cells transiently cotransfected with an ERα or ERβ expression plasmid and an estrogen-response element (ERE)-luciferase reporter plasmid. Bisphenol AF fully activated ERα (increasing activity to ~ 7 times the baseline level) in a dose-dependent manner at concentrations of 10^−10^ to 10^−5^ M (Figure 3A). The half-maximal effective concentration (EC_50_) of bisphenol AF was 58.7 nM.

When we compared potencies for ERα activation versus ERα binding to determine receptor activation potency [expressed as EC_50_ (nM)/IC_50_ (nM)], we found a clear discrepancy between 17β-estradiol and bisphenol AF. As shown in Table 3, we estimated the receptor activation potency for 17β-estradiol to be 0.085 (0.075 nM/0.88 nM based on values from Figure 3A and Table 1, respectively). In contrast, the receptor activation potency of bisphenol AF [1.099 (58.7 nM/53.4 nM)] was approximately 13 times greater than that of 17β-estradiol (Table 3). This means that the concentration of 17β-estradiol required to stimulate a 50% response is about 13 times lower than the concentration required to occupy 50% of receptors, whereas the concentration of bisphenol AF required to stimulate a 50% response is about the same as that required to occupy 50% of receptors. This suggests that the receptor conformation induced by bisphenol AF is not as conducive to receptor activation as that induced by 17β-estradiol when measured in HeLa cells.

BPA was an extremely weak activator of both ERα (EC_50_ = 317 nM) and ERβ (EC_50_ = 693 nM) based on the luciferase reporter gene assay. The receptor activation potencies of BPA for ERα (0.308) and ERβ (0.770) were 3.6 and 18.8 times greater than the receptor activation potencies of 17β-estradiol for ERα and ERβ, respectively (Table 3). These suggests that, compared with 17β-estradiol, the concentration of BPA required to stimulate a 50% response is much higher than the concentration required to occupy 50% of receptors. In addition, as shown in Figure 3B, BPA exhibited a reduced ability to bring about full activation of ERβ (3.5 times greater activity relative to baseline in response to BPA vs. an increase to 6 times the baseline level in response to 17β-estradiol). This difference in efficacy indicates that BPA does not have the same ability as 17β-estradiol to induce activation conformation when measured in HeLa cells on this promoter.

### Antagonist activity of bisphenol AF on ERβ

For ERβ, bisphenol AF was almost completely inactive, with very little increase in activity even at 10 μM, the highest concentration tested (Figure 3B). Based on the strong receptor-binding activity of bisphenol AF for ERβ (IC_50_ = 18.9 nM; Table 1), we expected that bisphenol AF would also have a high receptor activation potency for ERβ. This unexpected inactivity in the reporter gene assay suggests that bisphenol AF binding disrupts the ERβ-LBD activation conformation, in which the α-helix 12 (H12) of the receptor is normally positioned to recruit the coactivator protein conformation (Brzozowski et al. 1997; Ruff et al. 2000).

We therefore evaluated the antagonist activity of bisphenol AF against 17β-estradiol. When we examined 17β-estradiol, an endogenous agonist ligand of ERβ, in the presence of 0.01, 0.1, 1.0, and 10 μM bisphenol AF, its activity (EC_50_ = 0.075 nM) was gradually weakened. As shown in Figure 4A, the dose-dependent curves of 17β-estradiol shifted to the right with increasing concentrations of bisphenol AF, indicating that bisphenol AF effectively inhibits the interaction between 17β-estradiol and ERβ. When the results of Figure 4A were analyzed using a Schild plot, p*A*_2_, a measure of affinity of the antagonist for receptor, was calculated to be 7.87 from the dissociation equilibrium constant (*K*_B_ = 1.35 × 10^−8^ M).

The antagonist activity of bisphenol AF for 17β-estradiol/ERβ was further evidenced by assays in which we added serial concentrations of bisphenol AF (10^−12^ to 10^−5^ M) to a solution of 17β-estradiol maintained at a constant concentration. When 1 × 10^−8^ M 17β-estradiol was treated with bisphenol AF, the activity of 17β-estradiol was reduced in a dose-dependent manner in response to bisphenol AF concentrations ranging from 10^−10^ to 10^−5^ M (Figure 4B). We obtained a similar result for 1 × 10^−7^ M 17β-estradiol. These results demonstrate that bisphenol AF can antagonize the activity of 17β-estradiol on the ERβ receptor.

## Discussion

### Structural characteristics of bisphenols and ERs/ERRγ receptors

The differences in receptor selectivity between bisphenol AF and BPA are due to the CH_3_ ↔ CF_3_ substitution on the bisphenol backbone structure. Bisphenol AF is a hexafluoro derivative of BPA with the CH_3_ → CF_3_ substitution on the backbone structure of 2,2-disubstituted propane CH_3_–C–CH_3_. BPA binds strongly to ERRγ, but bisphenol AF binds to ERRγ only weakly; we therefore judged that the binding pocket of ERRγ-LBD possesses structural elements unfavorable for interaction with the trifluoro groups. The molecular size of CF_3_ is almost the same as that of CH_3_, and thus there would be no structural repulsion or steric hindrance between these groups. However, because the CF_3_ group is very electron rich, the structural elements standing face to face with CF_3_ must also be electron rich, resulting in their electrostatic repulsion.

In our previous study (Matsushima et al. 2007, 2008), we found that the ERRγ binding sites for BPA CH_3_ groups were Phe435 and Met306. Because the aromatic phenyl and S–CH_3_ groups of Phe435 and Met306 are electron rich, conditions would be unfavorable for binding of bisphenol AF’s electron-rich CF_3_ groups. Corresponding receptor residues in ERα are Leu525 and Leu384, respectively. Apparently, there would be no electrostatic repulsion between the bisphenol AF’s CF_3_ groups and the Leu residues. Such a release in structural stress must be very favorable for receptor activity and the selectivity of bisphenol AF for ERα.

In the present study, we found bisphenol AF to be a strong ligand for both ERα and ERβ receptors, although it shows a 3 times greater preference for ERβ over ERα. A much more important finding is that bisphenol AF functions in a different way for ERα and ERβ. Bisphenol AF is a full agonist for ERα but an antagonist for ERβ. The LBDs of ERα and ERβ share a high sequence identity (59%) and similar three-dimensional structures. We observed no obvious differences between ERα and ERβ in the ERE transcriptional assays in the presence of 17β-estradiol.

Among the amino acid residues lining the binding pockets of ERα and ERβ, two residues differ significantly: Leu384 in α-helix 5 (H5) of ERα is replaced by Met336 in ERβ, and Met421 in loop 6–7 of ERα is replaced by Ile373 in ERβ. These two residues are most probably responsible for the discriminative affinity and reverse functional activity of bisphenol AF for ERα and ERβ. Furthermore, because bisphenol AF is an ERβ antagonist, the binding of bisphenol AF to the ERβ ligand-binding pocket must damage the ERβ-LBD activation conformation, in which the α-helix 12 (H12) in LBD is positioned to recruit the coactivator proteins conformation (Brzozowski et al. 1997; Ruff et al. 2000). Bisphenol AF binding to LBDs of ERα and ERβ are being analyzed in light of the crystal structures in studies in progress in our laboratory.

### Bisphenol AF as a candidate of potential endocrine disruptor

Bisphenol AF is a potent estrogen agonist for ERα and a potent estrogen antagonist for ERβ. ERα and ERβ are widely distributed throughout the body, displaying distinct but overlapping expression patterns in a variety of tissues. ERα is expressed primarily in the uterus, liver, kidneys, and heart (Couse and Korach 1999), whereas ERβ is expressed primarily in the ovaries (Couse and Korach 1999), prostate (Couse and Korach 1999), lungs (Kuiper et al. 1997), and gastrointestinal tract and bladder (Nilsson et al. 2001). Coexpression of both receptors occurs in the mammary glands (Pettersson and Gustafsson 2001), epididymis (Pau et al. 1998), thyroid (Pau et al. 1998), adrenals (Pau et al. 1998), bone (Arts et al. 1997; Brandenberger et al. 1997), and certain regions of the brain (Couse and Korach 1999). [For additional information, see Nuclear Receptor Signaling Atlas (2010).] 17β-Estradiol plays a critical role in many physiological processes in both females and males. These include normal growth, development, and cell-type–specific gene regulation in tissues of the reproductive tract, central nervous system, and skeleton (Couse and Korach 1999; Nilsson et al. 2001; Pettersson and Gustafsson 2001). Bisphenol AF is a potent binder of ERα and ERβ and thus would perturb these physiological processes, perhaps providing significant adverse influences for the central and peripheral systems.

### Effects of the bisphenol trihalogenated methyl group on receptor actions

Bisphenol AF is an agonist for ERα and an antagonist for ERβ. Similar results have been reported for HPTE, a bisphenolic metabolite of methoxychlor [1,1,1-trichloro-2,2-bis(4-methoxyphenyl)ethane]. HPTE behaved as an ERα agonist and an ERβ antagonist with estrogen-responsive promoters in HeLa cells (Gaido et al. 1999). We confirmed these results in our assay systems as well. HPTE was a strong binder of ERα with IC_50_ = 59.1 nM and of ERβ with IC_50_ = 18.1 nM (Table 1). As reported previously by Gaido et al. (1999) and Nettles et al. (2004), HPTE acts as a full agonist for ERα but a strong antagonist for ERβ. However, bisphenol AF and HPTE differ in their receptor preference for ERRγ. HPTE was approximately 10 times more potent than bisphenol AF for ERRγ binding, although both chemicals were most strongly bound to ERβ (Tables 1, 2). As an antagonist for ERβ, bisphenol AF (p*A*_2_ = 7.87) was somewhat stronger than HPTE, the p*A*_2_ of which was reported to be 7.52 (Gaido et al. 1999). However, both bisphenol AF and HPTE are significantly potent as ERβ antagonists.

**Figure 1:**
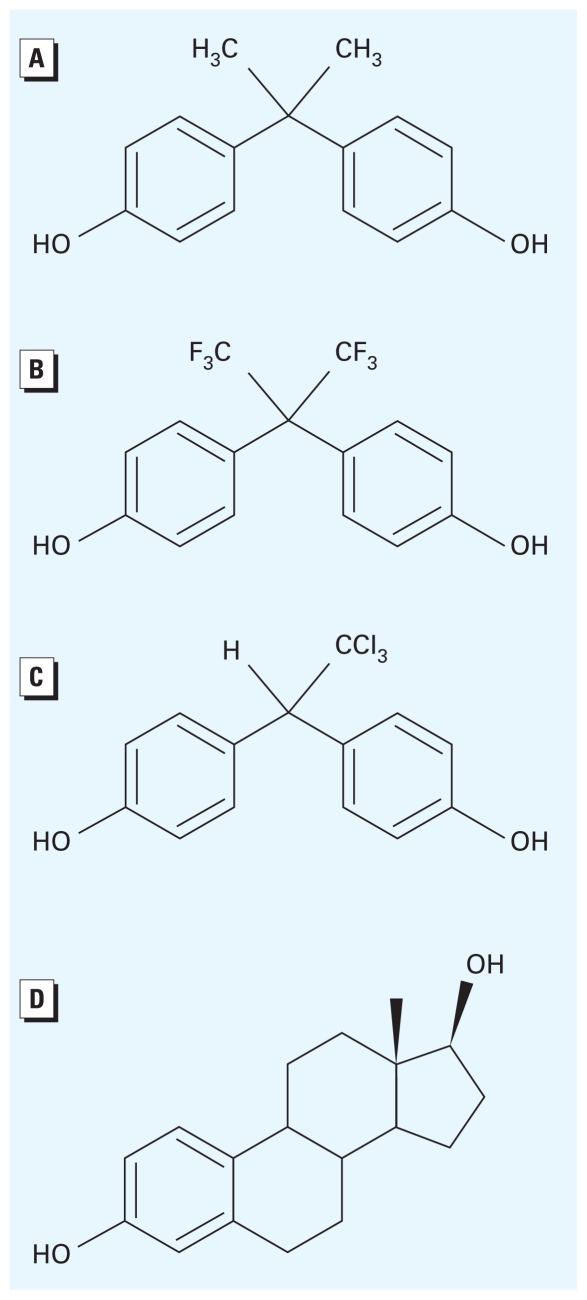
Chemical structures of (*A*) BPA, (*B*) bisphenol AF, (*C*) 2,2-bis(*p*-hydroxyphenyl)-1,1,1-trichloroethane (HPTE), and (*D*) 17β-estradiol.

**Figure 2:**
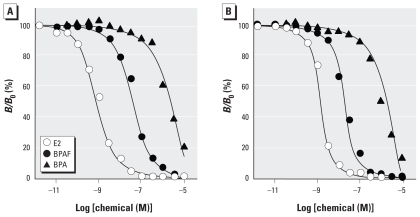
Radioligand receptor-binding assays of bisphenol AF (BPAF), BPA, and 17β-estradiol (E2) to measure the ability of the compounds to displace [^3^H]17β-estradiol in recombinant human ERα (*A*) and ERβ (*B*). *B*/*B*_0_, sample bound/maximum binding. The representative dose-dependent binding curves show the IC_50_ value closest to the mean IC_50_ from at least five independent assays. The IC_50_ values showed a between-experiment coefficient of variation of 5–12%.

**Figure 3:**
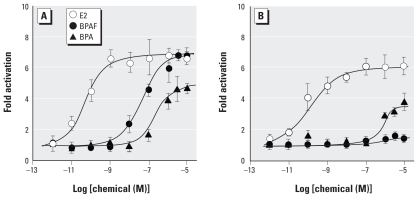
Luciferase-reporter gene assays of bisphenol AF (BPAF), BPA, and 17β-estradiol (E2) for ERα and ERβ using reporter gene (pGL3/3xERE) and either ERα or ERβ expression plasmid (pcDNA3/ERα or pcDNA3/ERβ) in HeLa cells. Concentration-dependent responses of 17β-estradiol, bisphenol AF, and BPA in the luciferase-reporter gene assay for ERα (*A*) and ERβ (*B*). For ERα, bisphenol AF displays full activation in a concentration-dependent manner, whereas for ERβ it displays extremely weak activity. 17β-Estradiol exhibits very strong activity, with approximately 4.5 times more activity induced at 10^−14^ to 10^−5^ M than at baseline.

**Figure 4:**
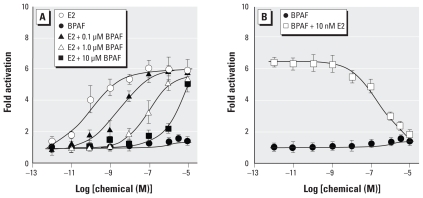
Effects of bisphenol AF (BPAF) on the agonist activity of 17β-estradiol (E2) in the luciferase-reporter gene assays for ERβ. (*A*) Concentration-dependent luciferase-reporter activities of 17β-estradiol by fold activation in the presence and absence of bisphenol AF (0.1, 1, or 10 μM); these concentrations of bisphenol AF clearly weaken the agonist activity of 17β-estradiol for ERβ. (*B*) Concentration-dependent effects of bisphenol AF on the agonist activity of 17β-estradiol; the agonist activity of 10 nM 17β-estradiol was clearly inhibited by bisphenol AF in a dose-dependent manner. Bisphenol AF itself sustained extremely weak activity for ERβ. In these assays, the reporter gene (pGL3/3xERE) and ERβ expression plasmid (pcDNA3/ERβ) were measured in HeLa cells.

Chemical structures of bisphenol AF and HPTE differ, with one of two CF_3_ groups of bisphenol AF replaced by CCl_3_ in HPTE, and the other by H (Figure 1). However, these compounds are similar in that both have trihalogenated methyl groups that may produce different activities for ERα and ERβ via their interactions with the ligand-binding pockets of each ER, namely, Leu384 in H5 of ERα ↔ Met336 in ERβ, and Met421 in loop 6–7 of ERα ↔ Ile373 in ERβ.

Methoxychlor is a chlorinated hydrocarbon pesticide structurally similar to DDT (dichlorodiphenyltrichloroethane) and thus is sometimes referred to as dimethoxy or methoxy DDT. It had been used to some degree as a replacement for DDT to protect crops, ornamentals, livestock, and pets against various insects, because it was believed to be metabolized more quickly than DDT, thus reducing or preventing bioaccumulation (Kapoor et al. 1970). Methoxychlor is uterotropic in the ovariectomized rat and can cause adverse developmental and reproductive effects in mice and rats (Alm et al. 1996; Cummings 1997; Hall et al. 1997). However, HPTE is approximately 100 times more active at ERs than is methoxychlor. To date, the use of methoxychlor has been banned in many countries, including the United States, Japan, and the European Union. All these issues clearly raise concerns that not only HPTE but also bisphenol AF may be a potential endocrine disruptor affecting either ERα or ERβ, or both.

## Conclusions

BPA binds strongly to ERRγ but very weakly to ERα and ERβ. In contrast, bisphenol AF binds very weakly to ERRγ but strongly to ERα and ERβ. These differences in receptor selectivity reflect subtle but distinct structural differences resulting from the CH_3_ ↔ CF_3_ substitution on the bisphenol backbone structure. The trifluoromethyl group is much more electronegative than the methyl group. These results suggest that apparently minor structural differences among chemicals and NRs may have pronounced effects on binding affinity and selectivity. Thus, the present study emphasizes the crucial importance of accurate evaluation of receptor responses to understanding interactions between endocrine-disrupting compounds and diverse human NRs. Taken together, these results clearly indicate the importance of examining the degree and ways in which bisphenol AF may influence the physiological roles of ERα and ERβ. Given that bisphenol AF and BPA function as endocrine disruptors, these chemicals would work differently via different NRs.

## Figures and Tables

**Table 1:** Receptor-binding characteristics of BPA and bisphenol AF for ERα, ERβ, and ERRγ.

	IC_50_ (nM)		
Compound	ERα	ERβ	ERRγ
17β-estradiol	0.88 ± 0.04	2.17 ± 0.12	NB
4-OHT	2.88 ± 0.15	3.17 ± 0.24	10.3 ± 0.8
BPA	1,030 ± 70	900 ± 70	9.70 ± 0.59
Bisphenol AF	53.4 ± 3.1	18.9 ± 0.84	358 ± 3.1
HPTE	59.1 ± 1.5	18.1 ± 1.9	36.4 ± 4.4

Abbreviations: HPTE, 2,2-bis(*p*-hydroxyphenyl)-1,1,1-trichloroethane; NB, not bound (no significant receptor binding at 10 μM, the highest concentration tested).

**Table 2:** Receptor-binding selectivity of BPA and AF for ERα, ERβ, and ERRγ.

	Receptor-binding selectivity			
Compound	ERα vs. ERβ	ERα vs. ERRγ	ERβ vs. ERRγ	Preferred receptor(s)
17β-estradiol	2.47 ERα	(ERα)^a^	(ERβ)^a^	ERα
4-OHT	1.10 ERα	3.58 ERα	3.25 ERβ	ERα ~ ERβ
BPA	1.14 ERβ	106.18 ERRγ	92.78 ERRγ	ERRγ
Bisphenol AF	2.83 ERβ	6.70 ERα	18.94 ERβ	ERβ
HPTE	3.27 ERβ	1.63 ERRγ	2.01 ERβ	ERβ

HPTE, 2,2-bis(*p*-hydroxyphenyl)-1,1,1-trichloroethane. Data are *n*-fold strength of the preferred receptor compared with the nonpreferred receptor; for example, “2.47 ERα” means that 17β-estradiol binds to ERα 2.47 times more strongly than to ERβ.

a Because of inactivity of 17β-estradiol in ERRγ, 17β-estradiol is active exclusively in ERα and ERβ.

**Table 3:** Binding affinities of 17β-estradiol, BPA, and bisphenol AF relative to their potencies for stimulating reporter gene activity by ERα and ERβ in HeLa cells.

	EC_50_ (nM)/IC_50_ (nM)	
Compound	ERα	ERβ
17β-estradiol	0.085 (1.0)	0.041 (1.0)
BPA	0.308 (3.6)	0.770 (18.8)
Bisphenol AF	1.099 (12.9)	—

Values in the parentheses show the relative value of the EC_50_/IC_50_ ratio (17β-estradiol = 1.0).
